# Fibroblast-associated TPM2 links cell-matrix remodeling to EMT-Notch signaling and gemcitabine resistance in intrahepatic cholangiocarcinoma

**DOI:** 10.1080/15384047.2026.2725344

**Published:** 2026-09-21

**Authors:** Yingjiao Zhu, Zhibin Qiao, Zhuhong Chen, Yahua Wu, Lirong Xiao, Fangyu Lin, Xiaoyan Lin, Xinli Wang

**Affiliations:** a Department of Medical Oncology, Fujian Medical University Union Hospital, Fuzhou, Fujian, People's Republic of China; b Fujian Medical University Union Clinical Medicine College, Fuzhou, Fujian, People's Republic of China; c Department of Cancer Institute, Fudan University Shanghai Cancer Center, Shanghai, People's Republic of China; d Department of Radiation Oncology, Fujian Medical University Union Hospital, Fuzhou, Fujian, People's Republic of China

**Keywords:** Intrahepatic cholangiocarcinoma, patient-derived organoids, TPM2, gemcitabine response, fibroblasts, cell-matrix remodeling

## Abstract

**Background:**

Intrahepatic cholangiocarcinoma (ICC) is an aggressive liver malignancy with heterogeneous responses to gemcitabine-based chemotherapy. The cellular and microenvironmental programs associated with the gemcitabine response remain incompletely understood. This study aimed to identify tumor microenvironment-associated regulators of gemcitabine response in ICC and evaluate their functional relevance using patient-derived organoids.

**Methods:**

Four public single-cell RNA sequencing datasets comprising 50,176 cells were integrated with TCGA-based transcriptomic and drug sensitivity analyses. High-dimensional weighted gene co-expression network analysis was used to identify cell type-associated modules. Predicted gemcitabine IC50-related differentially expressed genes were intersected with the fibroblast-associated module genes. Patient-derived ICC organoids were used for gemcitabine sensitivity testing and CRISPR/Cas9-mediated TPM2 knockout validation.

**Results:**

Single-cell analysis revealed fibroblast enrichment in ICC, and co-expression analysis identified fibroblast-associated modules enriched in extracellular matrix organization and ECM-receptor interaction. Integrated analysis identified TPM2 as a fibroblast-enriched candidate associated with poor prognosis, activation of epithelial-mesenchymal transition, TGF-*β* and Notch-related pathways, and dynamic cellular state transitions. Patient-derived ICC organoids retained features of matched tumors and showed distinct gemcitabine sensitivities that were broadly consistent with the clinical imaging responses. In a relatively gemcitabine-resistant organoid model, TPM2 knockout enhanced gemcitabine-induced cytotoxicity and suppressed EMT/ECM-related programs and Notch signaling.

**Conclusions:**

These findings identify TPM2-associated cell-matrix remodeling as a potential contributor to gemcitabine resistance in ICC and support patient-derived organoids as a functional platform for studying therapeutic vulnerabilities.

## Introduction

Intrahepatic cholangiocarcinoma (ICC) is an aggressive primary liver malignancy arising from the intrahepatic biliary epithelium that accounts for approximately 10–20% of primary hepatic malignancies.^1^ ^,^ ^2^ Its incidence has increased in many regions in recent decades, while early diagnosis remains challenging because symptoms are often nonspecific and tumors are frequently detected at an advanced stage.^1^ ^,^ ^3^ For patients with unresectable or metastatic biliary tract cancer, gemcitabine-based chemotherapy, particularly in combination with platinum agents, remains the major therapeutic backbone.^1^ ^,^ ^4^ ^,^ ^5^ However, clinical responses vary substantially among patients and intrinsic or acquired resistance frequently limits durable disease control. Therefore, defining the cellular and extracellular programs that shape the gemcitabine response is essential for improving preclinical modeling and therapeutic stratification in advanced ICC.

A prominent pathological feature of cholangiocarcinoma is its desmoplastic tumor microenvironment, characterized by abundant stromal cells, cancer-associated fibroblasts, and extracellular matrix (ECM) deposition.^6-8^ Cancer-associated fibroblasts remodel the ECM, alter tissue stiffness and cell-matrix interactions, and promote invasive tumor phenotypes through cytoskeletal remodeling, epithelial-mesenchymal transition (EMT)-associated programs, and paracrine signaling.^9-11^ These stromal and matrix-associated programs may also influence drug penetration, tumor cell plasticity, and chemotherapy tolerance. In addition, ECM remodeling and EMT-associated cellular plasticity can interact with developmental and survival-related signaling pathways, such as Notch signaling to support tumor adaptation under therapeutic stress.^12-15^ Despite the increasing recognition of fibroblast heterogeneity in ICC, the specific fibroblast-associated molecular programs that connect ECM remodeling, EMT-related signaling, and gemcitabine response remain incompletely defined.

Single-cell transcriptomics provides a powerful approach for dissecting cellular heterogeneity and cell-type-specific transcriptional programs within complex tumor ecosystems. Previous single-cell studies of ICC have revealed marked heterogeneity among malignant, immune, and stromal compartments and highlighted the importance of fibroblast populations and intercellular communication in shaping tumor behavior.^16^ ^,^ ^17^ However, single-cell discovery alone does not establish whether candidate stromal programs are functionally relevant to therapeutic response. Therefore, experimental models that preserve patient-specific tumor features are needed to test the relationship between candidate regulators and drug response.

Patient-derived organoids have emerged as clinically relevant three-dimensional models for disease modeling, drug screening, and personalized therapeutic evaluation.^18-21^ In biliary tract cancers, organoids can retain the key histological, molecular, and functional features of parental tumors and can be used to assess inter-patient heterogeneity in chemotherapy response.^22^ ^,^ ^23^ Thus, integrating single-cell stromal mapping with organoid-based drug testing may provide a useful framework for identifying tumor microenvironment-associated regulators of the gemcitabine response and for functionally evaluating their roles in tumor-intrinsic drug tolerance programs.

TPM2 encodes an actin-binding cytoskeletal protein involved in actin filament stabilization, contractility, and cytoskeletal organization.^24^ Cytoskeletal dynamics are closely linked to cell adhesion, migration, EMT, and ECM remodeling, processes that are central to tumor invasion and therapeutic adaptation.^25^ ^,^ ^26^ Given the close relationship between cytoskeletal remodeling, cell-matrix interactions, and EMT-associated signaling, TPM2 may represent a potential mediator linking fibroblast-enriched ECM programs with tumor cell plasticity and chemotherapy response. However, the role of TPM2 in ICC, particularly in relation to fibroblast-associated cell-matrix remodeling and gemcitabine response, remains unclear.

In this study, we investigated whether tumor microenvironment-associated cell-matrix programs are linked to gemcitabine response in ICC. To address this, we integrated public scRNA-seq datasets with TCGA-based bulk transcriptomic drug sensitivity analysis and patient-derived ICC organoid models. This strategy allowed us to identify candidate regulators associated with stromal remodeling and functionally assess TPM2 in an organoid-based gemcitabine response model.

## Materials and methods

### Patient-derived organoid samples

Patient-derived organoids were established from tumor specimens obtained from patients with stage IV ICC treated at Fudan University Shanghai Cancer Center. The use of human tumor specimens for organoid establishment was approved by the Ethics Committee of Fudan University Shanghai Cancer Center’s Review Board (protocol 050432-4-2307E), and written informed consent was obtained from all patients. Fresh tumor tissues were transferred on ice and processed for organoid culture within 24 h after collection.

### Tumor tissue processing

Tumor tissue processing was performed as previously described^27^ ^,^ ^28^ with minor modifications. Briefly, freshly obtained ICC tissues were minced into fragments of less than 1 mm^3^ and digested with 5 mg/mL collagenase type IV (#LS004188; Worthington) at 37 °C for 40–60 min. The resulting cell aggregates were filtered through a 100 μm cell strainer, washed twice with cold PBS, and centrifuged at 200 × g for 5 min. Cell pellets were resuspended in Matrigel (#356231; Corning), seeded into pre-warmed 24-well plates, and allowed to polymerize at 37 °C for 10 min. Complete organoid culture medium was then added, and cultures were maintained at 37 °C with medium changes every 3−4 days.

### Culture of ICC organoids

ICC organoids were cultured in complete organoid culture medium based on Advanced DMEM/F12 supplemented with growth factors, small molecules, and antibiotics as listed in Table 1. Organoids were passaged every 1–3 weeks according to their growth density using TrypLE Express (#12605028; Gibco).

**Table 1.** Composition of ICC organoid culture medium.

Component	Supplier	Final concentration
Advanced DMEM/F12	Gibco	Basal medium
Penicillin–Streptomycin	Gibco	1%
Primocin	InvivoGen	100 μg/mL
GlutaMAX	Gibco	1%
HEPES	Gibco	10 mM
B27 supplement	Gibco	1:50
N2 supplement	Gibco	1:100
*N*-acetylcysteine	Sigma	1.25 mM
R-spondin 1	Sino Biological Inc.	500 ng/mL
Noggin	Sino Biological Inc.	100 ng/mL
Nicotinamide	Sigma	10 mM
Gastrin	Sigma	10 nM
Recombinant human EGF	PeproTech	50 ng/mL
Recombinant human FGF10	PeproTech	100 ng/mL
HGF	PeproTech	25 ng/mL
Forskolin	Selleck	10 μM
Y-27632	Selleck	10 μM
A83-01	Selleck	5 μM

### Data acquisition

scRNA-seq datasets were obtained from the Gene Expression Omnibus (GEO) database, including GSE125449, GSE138709, GSE142784, and GSE181878. GSE125449 included 10 tumor samples, GSE138709 included 8 samples comprising 3 normal and 5 tumor samples, GSE142784 included 2 tumor samples, and GSE181878 included 2 samples comprising 1 normal and 1 tumor sample. Only samples with complete gene expression matrices were included for downstream analysis.

Bulk RNA-seq data of ICC were downloaded from TCGA, including 31 tumor tissues and 8 adjacent normal tissues from 39 distinct patients. Expression data were transformed into log2(TPM + 1) format for downstream analyzes. For analyzes integrating transcriptomic data with clinical survival information, one sample could not be matched to the corresponding clinical data and was therefore excluded, resulting in 38 samples available for overall survival analysis.

### Quality control and preprocessing of scRNA-seq data

All scRNA-seq datasets were processed using the Seurat R package (v4.0). Cells with fewer than 500 detected genes were excluded. Cells with extremely low or high UMI counts were removed based on sample-specific distributions. Cells with mitochondrial gene percentages greater than 20% were discarded. Cells with high ribosomal gene content were also excluded. Cells with high mitochondrial or ribosomal content were considered low-quality or stressed cells and were removed. After filtering, all datasets were merged, resulting in 50,176 high-quality cells retained for downstream analysis.

### Normalization, scaling, and dimensionality reduction

Gene expression data were normalized using the LogNormalize method, with each cell scaled to a total count of 10,000 followed by log transformation. Highly variable genes were identified using FindVariableFeatures, and the top 2,000 genes were selected for downstream analysis. Cell cycle scores were calculated using CellCycleScoring. ScaleData was applied to regress out mitochondrial gene percentage, ribosomal gene percentage, and cell cycle scores.

Principal component analysis (PCA) was performed based on highly variable genes. Batch effects across datasets were corrected using Harmony integration. UMAP was then used for nonlinear dimensionality reduction and visualization. Cell clustering was performed using the Louvain algorithm, resulting in 20 distinct clusters.

### Cell type annotation

Cell types were annotated based on canonical marker genes. T cells were annotated using CD3D and CD3E; epithelial cells using KRT18 and EPCAM; endothelial cells using CLDN5 and ENG; monocytes using FCN1; fibroblasts using COL1A1 and ACTA2; macrophages using C1QB and C1QA; dendritic cells using HLA-DPB1 and CD1C; plasma cells using MZB1 and IGJ; hepatocytes using APOA2 and APOC3; and B cells using CD79A and MS4A1. Annotation results were visualized using UMAP projections and dot plots.

### hdWGCNA analysis

High-dimensional weighted gene co-expression network analysis (hdWGCNA) was performed using the hdWGCNA R package. Only cells derived from tumor specimens were included in the hdWGCNA analysis, whereas cells from adjacent normal tissues were excluded. Genes expressed in at least 5% of cells were selected to construct the co-expression network. The soft-thresholding power was determined using the TestSoftPowers function, and *β* = 5 was selected to achieve scale-free topology. Hierarchical clustering was used to identify gene modules, which were visualized using dendrograms. A total of seven modules were identified, including turquoise, blue, green, black, red, yellow, and brown modules.

Module eigengenes were calculated using GetMEs, and correlations between module eigengenes and annotated cell types within the tumor-derived single-cell dataset were evaluated. The turquoise and blue modules, which showed strong correlations with fibroblasts, were selected for downstream analysis. The top 100 genes ranked by kME in each module were defined as hub module genes.

### Functional enrichment analysis

Functional enrichment analysis was performed using the clusterProfiler R package. Gene Ontology (GO) analysis was used to identify enriched biological processes. Kyoto Encyclopedia of Genes and Genomes (KEGG) analysis was performed to identify enriched pathways. Pathways with adjusted *P* values less than 0.05 were considered statistically significant.

### Drug sensitivity prediction

Drug sensitivity was predicted using the Genomics of Drug Sensitivity in Cancer (GDSC) database via the oncoPredict R package. The calcPhenotype function was applied to normalized TCGA expression data to estimate the IC50 of gemcitabine for each sample. Default parameters were used, including batch effect correction using ComBat, averaging expression values for duplicated genes, and filtering low-variance genes. Samples were stratified into high-IC50 (Hexp) and low-IC50 (Lexp) groups based on the median predicted IC50 value.

### Differential expression analysis

Differential gene expression analysis was performed using the limma R package (v3.58.1). Genes with *P* < 0.05 and |log2 fold change| >1 were considered differentially expressed. Results were visualized using volcano plots and heatmaps.

### GSVA and GSEA analysis

Gene set variation analysis (GSVA) was performed using the GSVA R package to estimate pathway activity across samples. Gene sets were obtained from the MSigDB Hallmark collection. For TPM2-related pathway analysis, samples were divided into TPM2-high and TPM2-low groups based on the median TPM2 expression level. Gene set enrichment analysis (GSEA) was performed to identify pathways differentially enriched between groups using MSigDB v7.0 gene sets. Pathways with adjusted *P* < 0.05 were considered statistically significant.

### Pseudotime trajectory analysis

Pseudotime trajectory analysis was conducted using the Monocle2 R package to reconstruct cellular transition trajectories. Cells used for trajectory analysis were ordered along a pseudotime axis based on transcriptional similarity. Differentially expressed genes along pseudotime were identified using the differentialGeneTest function. Trajectories were visualized by pseudotime-based coloring and state-based branch annotation. Genes dynamically expressed along pseudotime were clustered into groups based on expression patterns.

### Histology and immunofluorescence staining

Tissues and organoids were fixed in 4% paraformaldehyde, embedded in paraffin, and cut into 4 μm sections. H&E and immunofluorescence staining were performed according to standard protocols. Primary antibodies included AFP (#14550-1-AP; Proteintech; 1:200), CK19 (#GB15198; Servicebio; 1:600), ALB (#GB152080; Servicebio; 1:50), and Vimentin (#A11952SP; Abclonal; 1:100). DAPI (#D571; Invitrogen; 1:1000) was used for nuclear counterstaining. Images were acquired by brightfield or fluorescence microscopy.

### Organoid drug sensitivity assay

Organoid drug sensitivity assays were performed as previously described.^27^ ^,^ ^28^ Briefly, organoids were dissociated into small fragments and embedded in Matrigel in 96-well plates. After 24–48 h of recovery to allow the formation of three-dimensional organoid structures, organoids were treated with serial concentrations of gemcitabine. Cell viability was assessed after at least 72 h of drug exposure using CellTiter-Glo® 3D assay (#G9683, Promega) according to the manufacturer’s instructions. Dose-response curves were generated, and relative viability was normalized to vehicle-treated controls. Drug sensitivity was compared between organoid models derived from different ICC patients.

### CRISPR/Cas9-mediated TPM2 knockout in ICC organoids

CRISPR/Cas9-mediated gene editing in ICC organoids was performed as previously described.^28^ Briefly, two independent sgRNAs targeting TPM2 were cloned into the lentiCRISPR v2 vector using BsmBI sites and verified by Sanger sequencing. A non-targeting sgRNA was used as the control. The sgRNA oligonucleotide sequences are listed in Table 2.

**Table 2.** Oligonucleotide sequences for TPM2 knockout and RT-qPCR primers.

Application	Target	Forward sequence (5′–3′)	Reverse sequence (5′–3′)
CRISPR/Cas9 knockout	sgTPM2#1	CACCGTATTCTGAATCCGTGAAGG	AAACCCTTCACGGATTCAGAATAC
CRISPR/Cas9 knockout	sgTPM2#2	CACCGCGGCCCTAACCTGCTTGCAG	AAACCTGCAAGCAGGTTAGGGCCGC
RT-qPCR	NOTCH1	CACTGTGGGCGGGTCC	GTTGTATTGGTTCGGCACCAT
RT-qPCR	HES1	TCAACACGACACCGGATAAAC	GCCGCGAGCTATCTTTCTTCA
RT-qPCR	HEY1	ATCTGCTAAGCTAGAAAAAGCCG	GTGCGCGTCAAAGTAACCT
RT-qPCR	VIM	GACGCCATCAACACCGAGTT	CTTTGTCGTTGGTTAGCTGGT
RT-qPCR	SNAI1	TCGGAAGCCTAACTACAGCGA	AGATGAGCATTGGCAGCGAG
RT-qPCR	CDH2	TCAGGCGTCTGTAGAGGCTT	ATGCACATCCTTCGATAAGACTG
RT-qPCR	FN1	AGGAAGCCGAGGTTTTAACTG	AGGACGCTCATAAGTGTCACC
RT-qPCR	COL1A1	GAGGGCCAAGACGAAGACATC	CAGATCACGTCATCGCACAAC
RT-qPCR	GAPDH	GGAGCGAGATCCCTCCAAAAT	GGCTGTTGTCATACTTCTCATGG

Lentivirus was produced in HEK293T cells using psPAX2, pMD2.G, and PEI. ICCO2 organoids were dissociated into small clusters and transduced with concentrated lentivirus in the presence of polybrene for 4–6 h at 37 °C. After recovery in Matrigel with Y-27632-containing medium, edited organoid pools were selected with puromycin at 10 μg/mL for 3 days. TPM2 knockout efficiency was confirmed by western blotting.

### Western blotting

Western blotting of organoids was performed as previously described.^28^ Briefly, organoids were lysed in 1 × SDS loading buffer and denatured at 95 °C for 10 min. Equal volumes of lysates were separated by SDS-PAGE, transferred onto nitrocellulose membranes, and probed with antibodies against TPM2 (#A3096; ABclonal; 1:1000), Notch1 (#A19090; ABclonal; 1:1000), HES1 (#A0925; ABclonal; 1:1000) and *β*-Tubulin (#A12289; ABclonal; 1:5000). Signals were detected using HRP-conjugated secondary antibodies and enhanced chemiluminescence.

### Calcein-AM and PI live/dead staining

Calcein-AM/PI live/dead staining of organoids was performed as previously described.^27^ Briefly, control and TPM2-knockout organoids were treated with DMSO or 10 μM gemcitabine for 5 days. Organoids were gently washed twice with PBS and stained with Calcein-AM (#40719ES50; Yeasen) and PI (#40755ES64; Yeasen) according to the manufacturer’s instructions. Brightfield and fluorescence images were acquired under identical settings using an Olympus IX83 inverted microscope equipped with Zero Drift Compensation technology.

### RNA extraction and RT-qPCR

RT-qPCR was performed as previously described.^28^ Briefly, total RNA was isolated from control and TPM2-knockout ICCO2 organoids, and RNA quantity and purity were assessed using a NanoDrop spectrophotometer. cDNA was synthesized from 1 μg total RNA using HiScript IV All-in-One Ultra RT SuperMix for qPCR (Vazyme, R433-01). Quantitative PCR was performed using 2 × Taq Pro Universal SYBR qPCR Master Mix (Vazyme, Q713). Relative gene expression was normalized to GAPDH and calculated using the 2^−ΔΔCt method. Primer sequences are listed in Table 2.

### Quantification and statistical analysis

For the experimental assays, three independent biological replicates were performed unless otherwise specified. For organoid drug sensitivity assays, dose–response experiments were independently repeated three times. Calcein-AM/PI live/dead staining and organoid area quantification were performed in three independent biological experiments, with at least 10 and 20 organoids analyzed per group, respectively. For immunofluorescence quantification, three independent biological experiments were performed, with at least 20 fields of view analyzed per group in each experiment. RT-qPCR experiments were performed using three independent biological replicates, with three technical replicates for each biological replicate. Western blot analyzes were independently performed three times. Image quantification was performed using ImageJ or equivalent image analysis software. Data are presented as mean ± SEM unless otherwise indicated. Statistical analyzes were performed using GraphPad Prism or R software (version 4.3.0). Comparisons between two groups were performed using two-tailed Student’s t-test or Wilcoxon rank-sum test, as appropriate. Correlations were assessed using Pearson or Spearman correlation analysis. Survival analysis was conducted using the Kaplan–Meier method and compared using the log-rank test. All statistical tests were two-sided, and *P* < 0.05 was considered statistically significant.

## Results

### Single-cell transcriptomic landscape reveals fibroblast enrichment and active tumor-associated programs in ICC

To characterize the tumor microenvironment of ICC, we integrated four public scRNA-seq datasets, including GSE125449, GSE138709, GSE142784, and GSE181878. After quality control, cells with fewer than 500 detected genes were excluded. A total of 50,176 high-quality cells were retained for downstream analysis **(Figure S1A−B)**. We then identified 2,000 highly variable genes and performed normalization, dimensionality reduction, and batch correction using Harmony **(Figure S1C−F)**.

**Figure 1.**
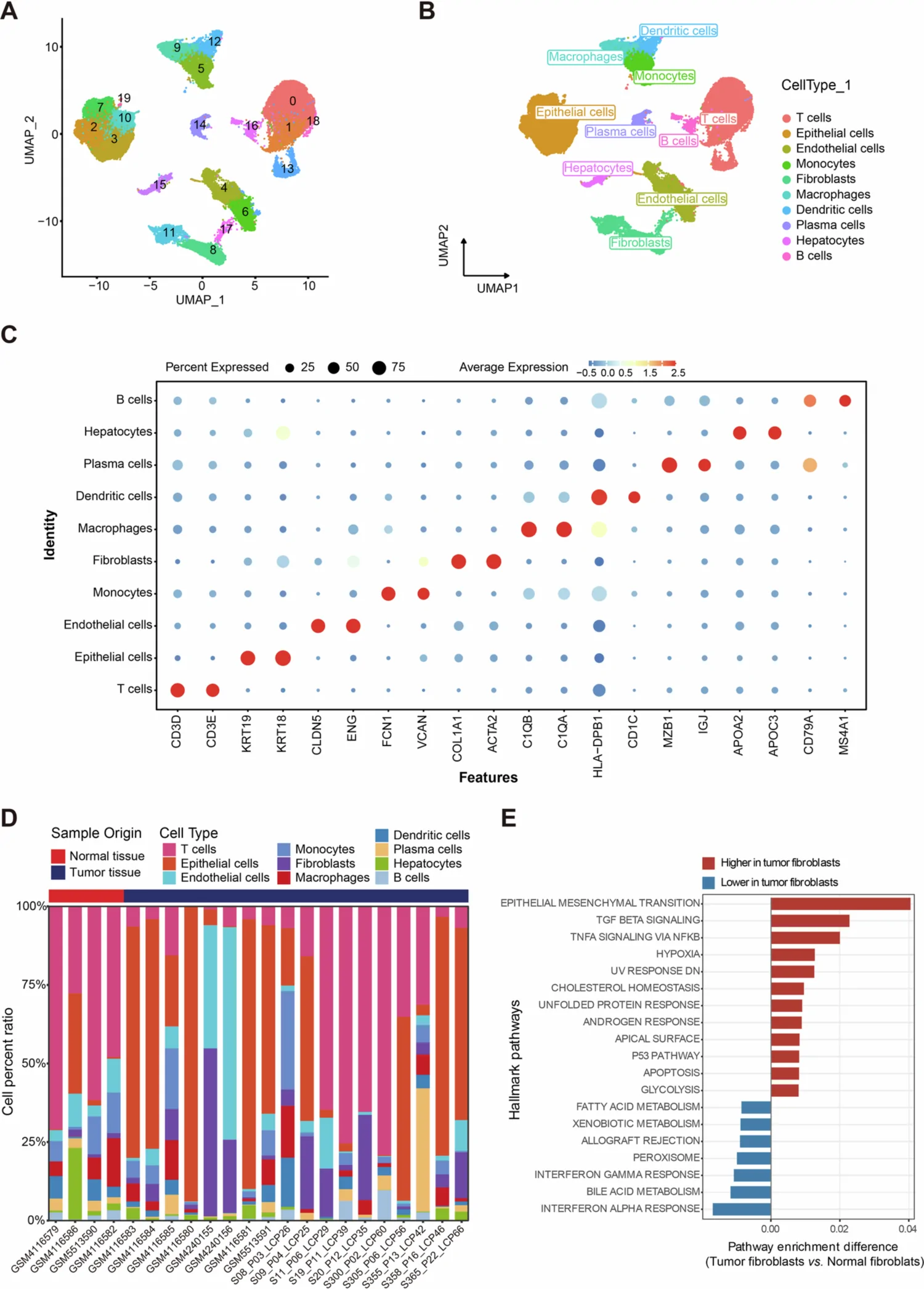
Single-cell landscape and cell-type-specific transcriptional programs in intrahepatic cholangiocarcinoma. (A) UMAP visualization of single cells after dimensionality reduction, showing 20 transcriptionally distinct clusters. (B) Annotation of the 20 clusters into 10 major cell populations, including T cells, epithelial cells, endothelial cells, monocytes, fibroblasts, macrophages, dendritic cells, plasma cells, hepatocytes, and B cells. (C) Dot plot showing the expression of canonical marker genes across annotated cell types. Dot size represents the proportion of expressing cells, and color intensity indicates average expression. (D) Stacked bar plot showing the relative proportions of annotated cell types in each individual sample. The annotation bar above indicates the sample origin. **(E)** Differential HALLMARK pathway activity between tumor-derived and adjacent normal-derived fibroblasts. Bar plot showing the top differentially enriched MSigDB HALLMARK pathways based on AUCell scores in annotated fibroblasts (adjacent normal, 123 cells; tumor, 4,060 cells). The x-axis represents the mean difference in pathway activity between tumor and adjacent normal fibroblasts, with positive and negative values indicating higher and lower activity in tumor-derived fibroblasts, respectively. Red and blue bars indicate pathways with significantly higher and lower activity, respectively, in tumor-derived fibroblasts after Benjamini-Hochberg false discovery rate correction.

Uniform manifold approximation and projection (UMAP) analysis identified 20 transcriptionally distinct clusters (Figure 1A). Based on canonical marker genes, these clusters were annotated into 10 major cell types, including T cells, epithelial cells, endothelial cells, monocytes, fibroblasts, macrophages, dendritic cells, plasma cells, hepatocytes, and B cells (Figure 1B−C). Sample-level analysis revealed substantial heterogeneity in cellular composition across individual tumor and adjacent normal specimens (Figure 1D). Further examination of sample and tissue origin showed that fibroblasts were strongly represented in tumor tissues: among the fibroblast cells captured in the integrated dataset, approximately 97% were derived from tumor samples compared with 3% from adjacent normal samples, with a significant difference in tissue-origin distribution **(Figure S2A−B**; Pearson’s chi-squared test, *P* < 0.0001**)**. These findings supported a prominent association of fibroblasts with the ICC tumor microenvironment.

We next specifically compared HALLMARK pathway activity between tumor-derived and adjacent normal-derived fibroblasts using AUCell. Tumor-derived fibroblasts exhibited higher activity of several cancer-associated programs, most prominently epithelial-mesenchymal transition, TGF-*β* signaling, TNFα signaling via NF-κB, and hypoxia-related pathways (Figure 1E). These results indicate tumor-associated functional reprogramming of fibroblasts and further support their potential contribution to ICC progression. We therefore focused subsequent analyzes on fibroblast-associated transcriptional programs.

### hdWGCNA identifies fibroblast-associated co-expression modules enriched in extracellular matrix remodeling

**Figure 2.**
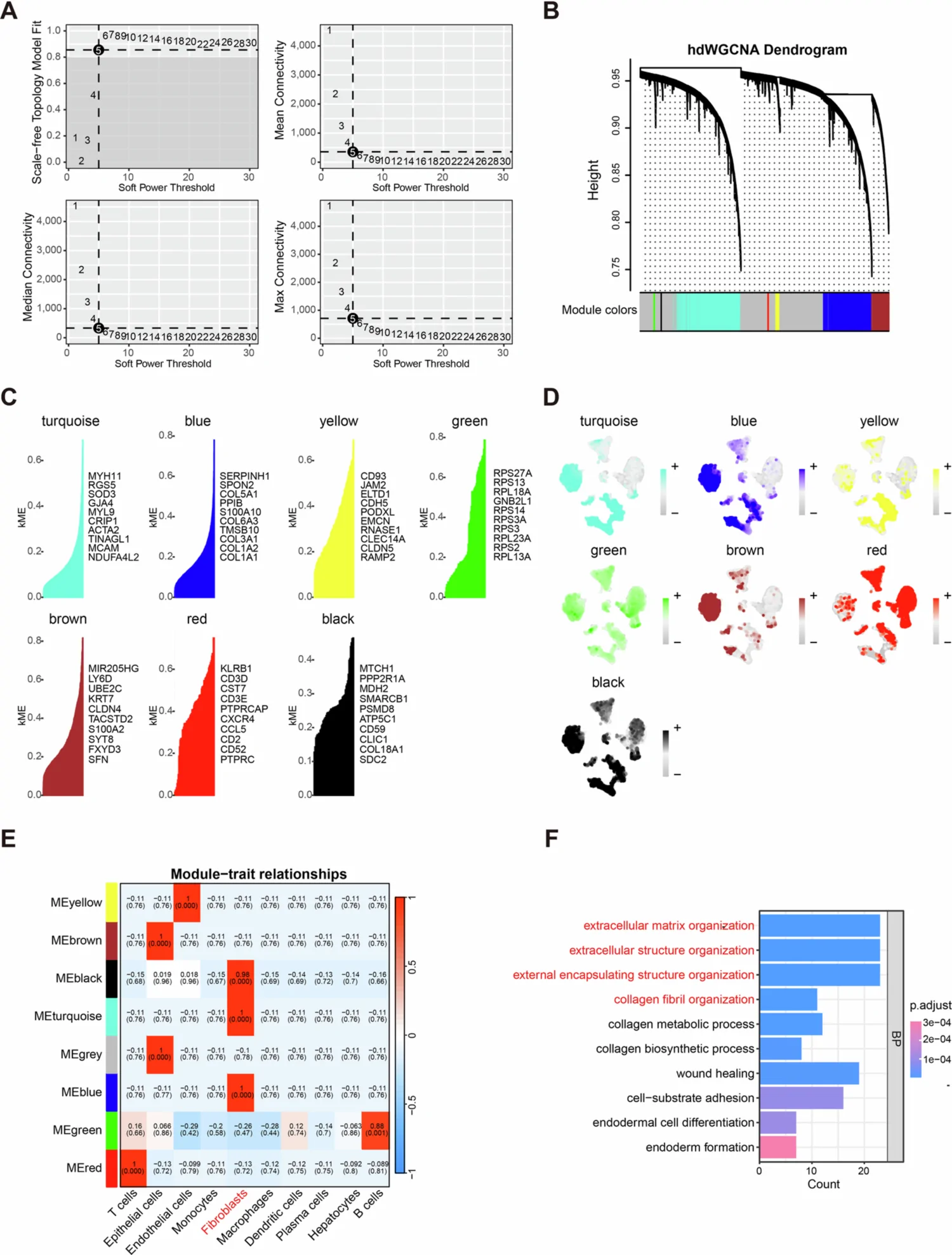
Identification of fibroblast-associated co-expression modules by hdWGCNA. (A) Determination of the soft-thresholding power for network construction. Scale-free topology fit index and connectivity metrics are shown across candidate thresholds. (B) Hierarchical clustering dendrogram of genes based on topological overlap, with modules indicated by distinct colors. (C) Representative hub genes ranked by module membership (kME) in each module. (D) UMAP visualization showing module activity scores across single cells. (E) Correlation heatmap between module eigengenes and annotated cell types. Color represents Pearson correlation coefficients. (F) Gene Ontology biological process enrichment analysis of fibroblast-associated module genes.

To identify gene modules associated with fibroblast-related programs, we performed hdWGCNA using only cells derived from tumor specimens. A soft-thresholding power of 5 was selected to construct the co-expression network (Figure 2A). This analysis identified seven gene modules (Figure 2B).

Module activity and eigengene correlation analyzes showed that the turquoise and blue modules were most strongly associated with fibroblasts (Figure 2C−E). Based on module membership scores, the top 100 genes from each of these two modules were selected as candidate fibroblast-associated genes for further analysis.

Functional enrichment analysis showed that these module genes were mainly enriched in ECM-related biological processes, including extracellular matrix organization, extracellular structure organization, and external encapsulating structure organization (Figure 2F). KEGG pathway analysis also revealed enrichment of the ECM-receptor interaction pathway **(Figure S3)**. Together, these findings identify fibroblast-associated co-expression programs within tumor tissues that are closely linked to ECM remodeling in ICC.

### Integration of predicted gemcitabine sensitivity and co-expression analysis identifies fibroblast-associated candidate genes

**Figure 3.**
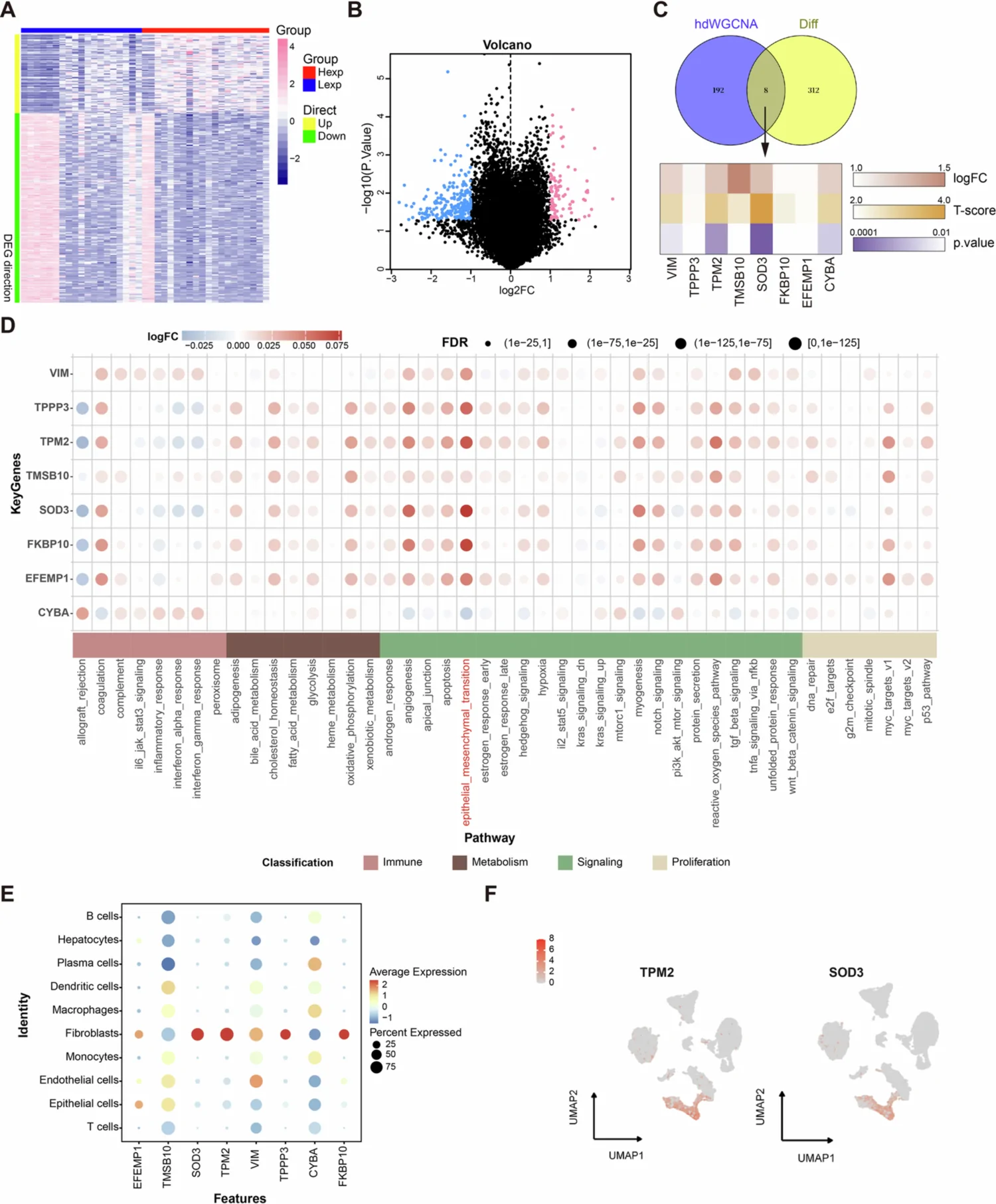
Identification of key genes associated with predicted gemcitabine sensitivity in intrahepatic cholangiocarcinoma. (A) Heatmap of differentially expressed genes between the high predicted gemcitabine IC50 (Hexp) and low predicted gemcitabine IC50 (Lexp) groups, stratified by the median predicted IC50. Upregulated genes show higher expression in Hexp than in Lexp samples, whereas downregulated genes show the opposite pattern. Red and blue indicate higher and lower relative expression, respectively. (B) Volcano plot of differentially expressed genes identified using limma. Positive log2 fold changes indicate higher expression in Hexp relative to Lexp. Upregulated genes are shown in red, downregulated genes in blue, and non-significant genes in black. (C) Venn diagram showing the overlap between hdWGCNA-derived hub genes and differentially expressed genes. The heatmap below summarizes the log fold change, T score, and *P* value of the overlapping candidate genes. (D) Bubble plot showing associations between candidate genes and pathway activity scores across immune, metabolic, signaling, and proliferation-related pathways. Color represents log fold change, and dot size reflects false discovery rate. (E) Dot plot showing the expression of candidate genes across major cell types. Dot size indicates the proportion of expressing cells, and color intensity represents average expression level. (F) Feature plots showing the distribution of TPM2 and SOD3 across the UMAP embedding.

To explore genes associated with gemcitabine response, we predicted gemcitabine sensitivity in TCGA ICC samples using the GDSC database and the oncoPredict algorithm. Samples were divided into high-predicted-IC50 (Hexp) and low-predicted-IC50 (Lexp) groups according to the median predicted IC50 value. Differential expression analysis identified 320 genes between the two groups, including 94 upregulated and 226 downregulated genes in the Hexp group (Figure 3A−B).

We then intersected these differentially expressed genes with the 200 fibroblast-associated module genes identified by hdWGCNA. This analysis identified eight overlapping genes (Figure 3C), which were considered candidate genes potentially linking fibroblast-associated programs with gemcitabine response.

Pathway analysis showed that several candidate genes, including VIM, TPPP3, TPM2, SOD3, FKBP10, and EFEMP1, were associated with EMT and related signaling pathways. In contrast, TMSB10 was linked to oxidative phosphorylation, while CYBA was linked to immune-related pathways (Figure 3D).

We further examined the single-cell expression patterns of the eight candidate genes. Although TPPP3 and FKBP10 also exhibited fibroblast-enriched expression, TPM2 and SOD3 showed the most pronounced fibroblast-preferential patterns, characterized by higher average expression and a greater proportion of expressing cells within the fibroblast population (Figure 3E−F and **Figure S4)**. Together with their associations with EMT-related pathways and predicted gemcitabine response, these findings prioritized TPM2 and SOD3 as the leading fibroblast-associated candidates for subsequent analyzes.

### TPM2 is associated with poor prognosis, pro-tumorigenic signaling, and pseudotime dynamics in ICC

To further evaluate the clinical relevance of candidate genes, expression levels were examined in TCGA datasets. Both TPM2 and SOD3 were significantly upregulated in tumor tissues compared with adjacent normal tissues **(Figure S5A)**, with TPM2 showing a more pronounced difference.

**Figure 4.**
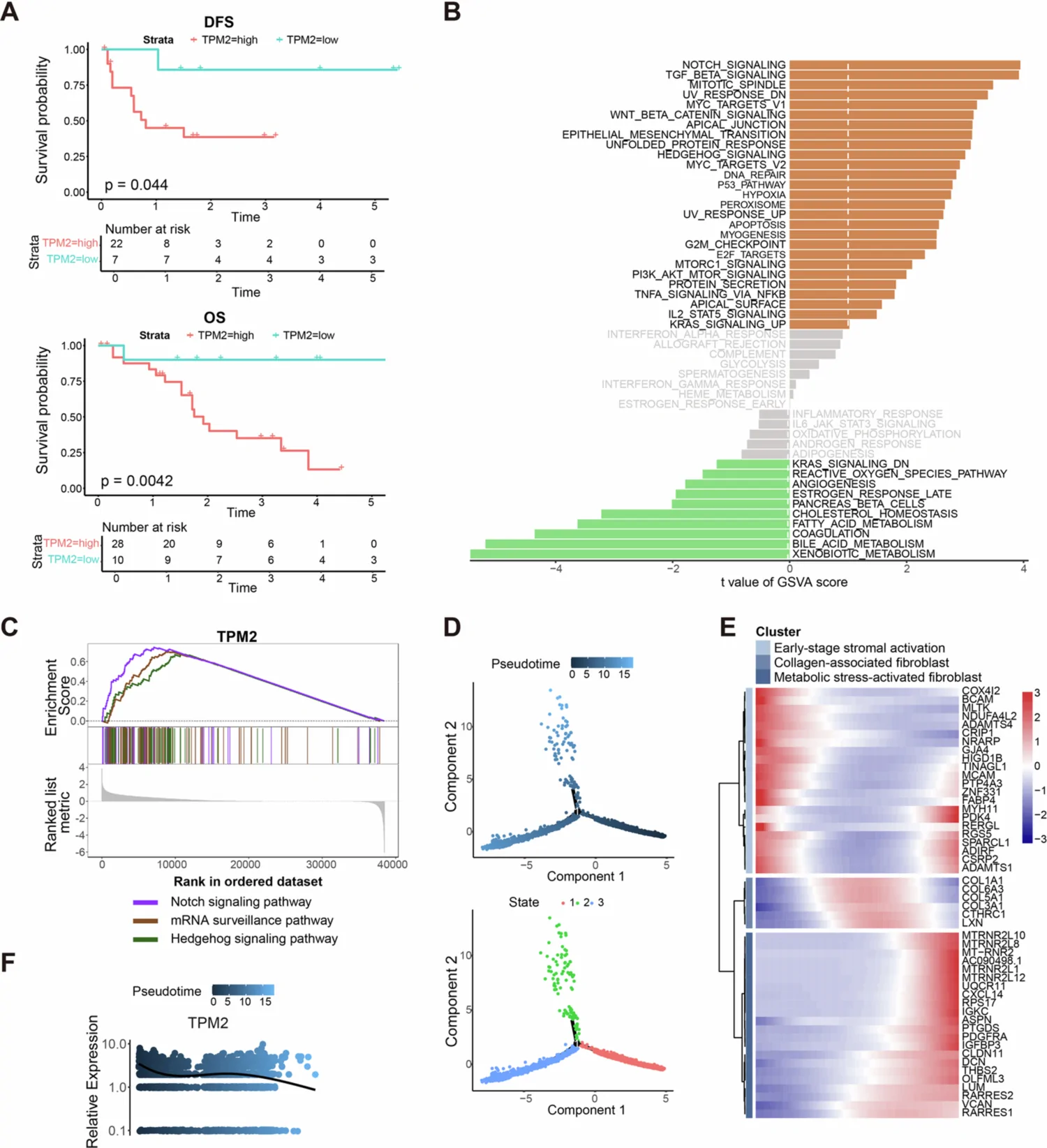
TPM2 is associated with poor prognosis, pathway activation, and dynamic cellular transitions in intrahepatic cholangiocarcinoma. (A) Kaplan-Meier survival curves for disease-free survival (DFS) and overall survival (OS) comparing TPM2-high and TPM2-low groups. (B) GSVA showing pathways differentially enriched between TPM2-high and TPM2-low groups based on Hallmark gene sets. Orange bars indicate pathways enriched in the TPM2-high group, green bars indicate pathways enriched in the TPM2-low group, and gray bars indicate non-significant or less prominent pathways. (C) GSEA plots showing representative pathways associated with TPM2 expression, including Notch signaling, mRNA surveillance, and Hedgehog signaling. (D) Pseudotime trajectory analysis using Monocle2. The upper panel shows cells colored by pseudotime, and the lower panel shows cells colored by inferred cell state. (E) Heatmap of genes dynamically regulated along pseudotime. Genes are grouped into three clusters according to their temporal expression patterns. (F) Expression dynamics of TPM2 along pseudotime, showing an overall decreasing trend during cellular progression.

Survival analysis revealed that high TPM2 expression was significantly associated with worse disease-free survival (DFS) and overall survival (OS) (Figure 4A). In contrast, SOD3 showed less consistent prognostic associations. Patients with high SOD3 expression showed better DFS, whereas OS did not differ significantly between the SOD3-high and SOD3-low groups **(Figure S5B)**. Based on its consistent association with poor prognosis, TPM2 was selected for further analysis.

We next performed GSVA to explore pathways associated with TPM2 expression. TPM2-high tumors were enriched in EMT, TGF-*β* signaling, Notch signaling, Wnt signaling, cell cycle, and DNA repair-related pathways. In contrast, metabolic pathways, including fatty acid metabolism, bile acid metabolism, and xenobiotic metabolism, were reduced in TPM2-high tumors (Figure 4B). These results suggest that high TPM2 expression is associated with a stromal- and EMT-activated transcriptional state. GSEA further showed enrichment of Notch signaling, mRNA surveillance, and Hedgehog signaling pathways in TPM2-high tumors (Figure 4C).

We then used Monocle2 to perform pseudotime trajectory analysis. The inferred trajectory revealed distinct cellular states along the pseudotime axis (Figure 4D), with gene expression programs dynamically changing along the inferred trajectory. Genes such as COX4I2 and BCAM showed higher expression toward the early portion of the inferred pseudotime trajectory, whereas genes including MTRNR2L10 and MTRNR2L8 showed higher expression toward the later portion (Figure 4E). TPM2 expression showed an overall decreasing trend along the inferred pseudotime trajectory (Figure 4F), suggesting that TPM2 expression varies across distinct transcriptional states. In contrast, SOD3 showed a non-linear pattern, with an initial decrease followed by an increase at later pseudotime points (**Figure S5C**). Together with the survival and pathway results, these findings support TPM2 as the main candidate gene for further functional analysis.

### Patient-derived ICC organoids support the functional role of TPM2 in gemcitabine response

**Figure 5.**
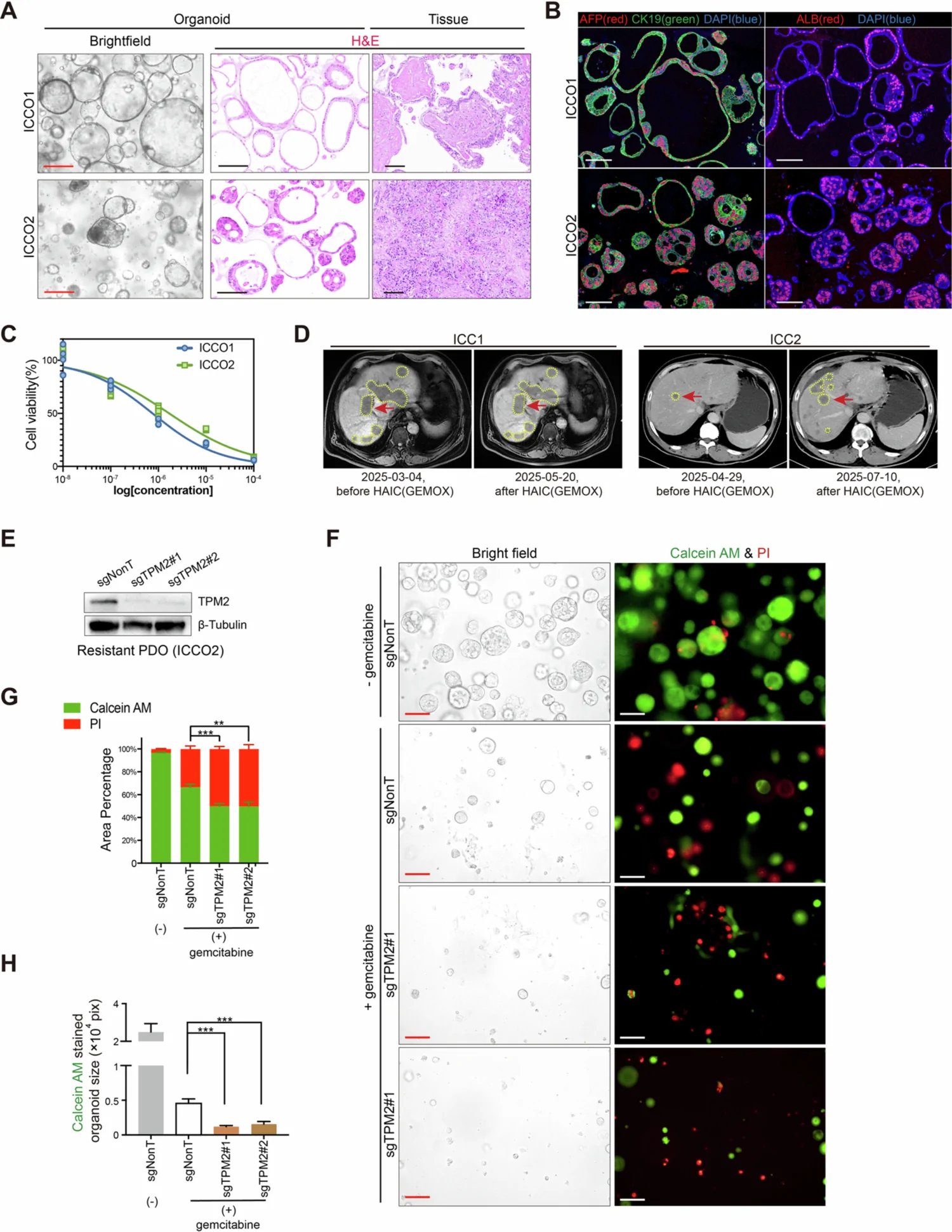
Functional validation of TPM2-associated gemcitabine response in patient-derived intrahepatic cholangiocarcinoma organoids. (A) Representative brightfield images of patient-derived intrahepatic cholangiocarcinoma organoids and hematoxylin and eosin (H&E) staining of organoids and matched primary tumor tissues from two cases. Scale bars, 100 μm. (B) Representative immunofluorescence staining showing AFP (red) and CK19 (green) in organoids (left panels), and ALB (red) in corresponding organoid samples (right panels). Nuclei are counterstained with DAPI (blue). Scale bars, 100 μm. (C) Dose-response curves showing gemcitabine sensitivity of two intrahepatic cholangiocarcinoma organoid models. Cell viability was assessed after treatment with serial concentrations of gemcitabine. Data are representative of three independent biological experiments. (D) Representative radiologic images obtained before treatment and after two cycles of hepatic arterial infusion chemotherapy (HAIC) with gemcitabine plus oxaliplatin (GEMOX) from the patients corresponding to ICCO1 and ICCO2 organoids. Yellow dashed outlines delineate the radiologically visible intrahepatic tumor lesions, and yellow arrows highlight the corresponding lesions to facilitate comparison of tumor changes before and after treatment. (E) Representative western blot validation of TPM2 knockout in the relatively gemcitabine-resistant ICCO2 organoid model using two independent sgRNAs targeting TPM2. *β*-Tubulin was used as a loading control. Data are representative of three independent biological experiments. (F) Representative brightfield images and corresponding Calcein-AM/PI fluorescence images of control and TPM2-knockout ICCO2 organoids following 5-day treatment with DMSO or 10 μM gemcitabine. Calcein-AM marks viable cells, whereas PI marks dead cells. Scale bars, 50 μm. (G) Quantification of Calcein-AM-positive and PI-positive areas in ICCO2 organoids following 5-day treatment with DMSO or 10 μM gemcitabine. At least 10 organoids per group were quantified in each independent biological experiment. Data are presented as mean ± SEM from three independent biological experiments. (H) Quantification of Calcein-AM-positive organoid area following 5-day treatment with DMSO or 10 μM gemcitabine. At least 20 organoids per group were quantified in each independent biological experiment. Data are presented as mean ± SEM from three independent biological experiments. *P < 0.05, **P < 0.01, ***P < 0.001.

To evaluate the role of TPM2 in a clinically relevant model, we established patient-derived organoids, named ICCO1 and ICCO2, from two patients with advanced ICC. The clinical characteristics of these patients are summarized in Table 3. Both organoid models formed typical three-dimensional cystic or compact structures in Matrigel culture (Figure 5A). Hematoxylin and eosin (H&E) staining showed that the organoids retained key histological features of their matched primary tumor tissues (Figure 5A). We next examined lineage-associated markers in these organoids. Immunofluorescence staining showed CK19 expression in both organoid models, supporting cholangiocytic differentiation. AFP and ALB signals were more pronounced in ICCO2 than in ICCO1, indicating stronger hepatobiliary lineage marker expression in ICCO2 (Figure 5B).

**Table 3.** Clinical characteristics of ICC patients used for organoid establishment.

Patient	Age (years)	Sex	Clinical stage	Viral infection	Histological type	Liver cirrhosis	Distant metastases	Treatment information
ICC1	58	Male	T4NxM1, stage IV	None	Poorly differentiated adenocarcinoma	Absent	Pulmonary metastasis	GEMOX HAIC; radiologic assessment after 2 cycles; 7 treatments in total (Mar 2025–Mar 26, 2026); subsequently lost to follow-up.
ICC2	55	Male	T4N3M1, stage IV	None	Poorly differentiated adenocarcinoma	Absent	Bilateral pulmonary, intrahepatic, peritoneal, bone metastases	GEMOX HAIC; radiologic assessment after 2 cycles; subsequently switched to TACE with an AG regimen in Aug 2025.

Abbreviations: GEMOX HAIC, gemcitabine plus oxaliplatin-based hepatic arterial infusion chemotherapy; TACE, transarterial chemoembolization; AG, nab-paclitaxel plus gemcitabine.

Note: Tumor tissues for organoid establishment were collected before the initiation of GEMOX HAIC. For GEMOX HAIC, both patients received gemcitabine (1.8 g) plus oxaliplatin (150 mg) via hepatic arterial infusion per treatment. The radiologic images presented in this study were obtained after two cycles of GEMOX HAIC in both patients.

We then performed dose-response assays using serial concentrations of gemcitabine. ICCO1 and ICCO2 displayed differential gemcitabine sensitivity, with ICCO2 showing lower sensitivity than ICCO1 (Figure 5C; IC50, ICCO1 vs. ICCO2: 7.541 × 10^^−7^ M vs. 1.569 × 10^^−6^ M**)**. Notably, the tumor tissues used to establish ICCO1 and ICCO2 were collected before the initiation of hepatic arterial infusion chemotherapy (HAIC) with gemcitabine plus oxaliplatin (GEMOX). To explore whether organoid-based drug response was broadly consistent with the corresponding clinical treatment response, we examined radiologic images obtained before treatment and after two cycles of GEMOX HAIC, with gemcitabine (1.8 g) plus oxaliplatin (150 mg) administered via hepatic arterial infusion. In line with the organoid drug-sensitivity results, the patient from whom ICCO2 was derived showed more evident radiologic tumor progression after two cycles of GEMOX HAIC, whereas the patient from whom ICCO1 was derived exhibited relatively stable disease on imaging (Figure 5D). These findings suggest that patient-derived ICC organoids may capture inter-patient heterogeneity in gemcitabine response and provide a clinically relevant platform for functional drug-response testing. Therefore, ICCO2 was selected as a relatively gemcitabine-resistant model for subsequent TPM2 knockout experiments to determine whether TPM2 depletion could enhance gemcitabine sensitivity, whereas the already sensitive ICCO1 model provided a more limited dynamic range for detecting further sensitization.

To test whether TPM2 contributes to gemcitabine response, we generated TPM2-knockout ICCO2 organoids using two independent sgRNAs. Western blotting confirmed efficient TPM2 depletion in both sgTPM2#1 and sgTPM2#2 organoids compared with non-targeting control organoids (Figure 5E). Control and TPM2-knockout organoids were then treated with DMSO or 10 μM gemcitabine for 5 days, and cell viability was assessed using Calcein-AM/PI live/dead staining. Under DMSO treatment, control organoids maintained clear viable structures. Gemcitabine treatment reduced organoid viability, and this effect was markedly enhanced by TPM2 knockout, as indicated by decreased Calcein-AM-positive viable areas and increased PI-positive dead-cell signals (Figure 5F−G; Calcein-AM-positive area proportion, sgNonT vs. sgTPM2#1 vs. sgTPM2#2: 66.61 ± 2.61% vs. 50.06 ± 2.31% vs. 49.73 ± 3.82%; sgNonT vs. sgTPM2#1, *P* = 0.0002; sgNonT vs. sgTPM2#2, *P* = 0.0018**)**. Quantitative analysis further confirmed that TPM2 depletion significantly reduced the Calcein-AM-positive organoid area under gemcitabine treatment (Figure 5H; sgNonT vs. sgTPM2#1 vs. sgTPM2#2: 4551 ± 668.8 pixels vs. 1218 ± 167.1 pixels vs. 1591 ± 361.8 pixels; sgNonT vs. sgTPM2#1, *P* < 0.0001; sgNonT vs. sgTPM2#2, *P* = 0.0004**)**. Together, these results suggest that TPM2 contributes to gemcitabine tolerance in patient-derived ICC organoids and that TPM2 depletion sensitizes relatively resistant organoids to gemcitabine-induced cytotoxicity.

### TPM2 depletion suppresses EMT/ECM-related programs and Notch signaling in ICC organoids

**Figure 6.**
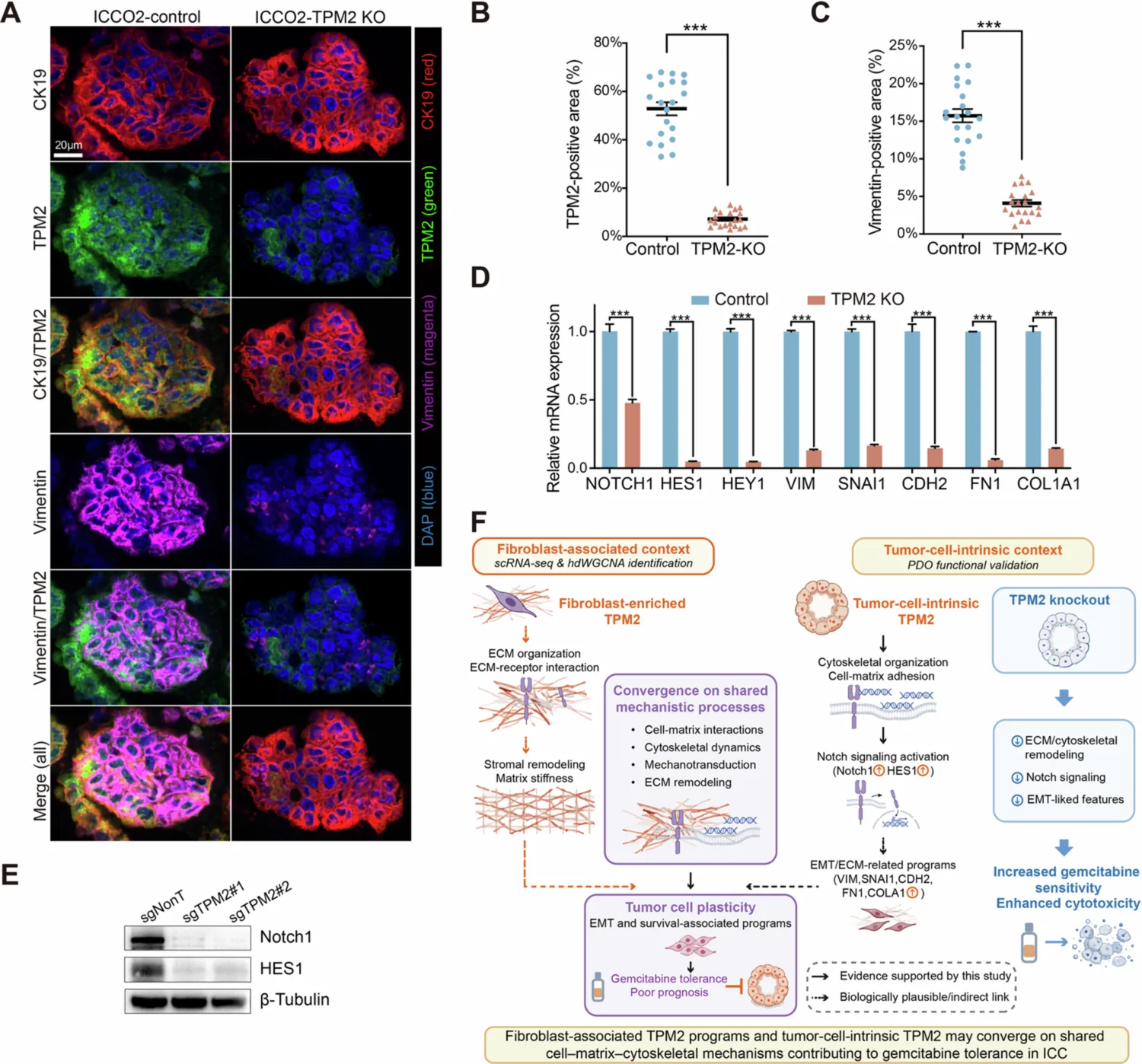
TPM2 depletion suppresses EMT/ECM-related programs and Notch signaling in intrahepatic cholangiocarcinoma organoids. (A) Representative immunofluorescence staining of CK19, TPM2, and Vimentin in control and TPM2-knockout ICCO2 organoids. CK19 marks cholangiocytic epithelial tumor cells, TPM2 denotes the knockout target, and Vimentin serves as an EMT-associated marker. Nuclei were counterstained with DAPI. Scale bar, 20 μm. (B, C) Quantification of TPM2-positive area (B) and Vimentin-positive area (C) in control and TPM2-knockout ICCO2 organoids. At least 20 fields of view per group were quantified in each independent biological experiment. Positive areas were quantified within organoid regions using ImageJ under identical imaging and thresholding settings. Data are presented as mean ± SEM. ***P < 0.001. (D) RT-qPCR analysis of Notch pathway genes, EMT-associated markers, and ECM-related genes in control and TPM2-knockout ICCO2 organoids. Relative mRNA expression was normalized to GAPDH and is shown relative to the control group. Experiments were performed using three independent biological replicates, with three technical replicates per biological replicate. (E) Representative western blot analysis showing reduced Notch1 and HES1 expression after TPM2 knockout in ICCO2 organoids. *β*-Tubulin was used as a loading control. Data are representative of three independent biological experiments. (F) Proposed model illustrating the convergence of fibroblast-associated and tumor-cell-intrinsic TPM2 programs on cell-matrix/cytoskeletal remodeling, Notch-EMT signaling, and gemcitabine tolerance. Solid arrows indicate relationships directly supported by the experimental data in the corresponding context, whereas dashed arrows indicate inferred, indirect, or biologically plausible relationships that require further validation.

To further explore the molecular programs associated with TPM2-mediated gemcitabine tolerance, we examined EMT/ECM-related features in TPM2-knockout ICCO2 organoids. Immunofluorescence staining showed that CK19 expression was retained in both control and TPM2-knockout organoids, supporting the preservation of cholangiocytic epithelial tumor features after TPM2 depletion. In contrast, TPM2 staining was markedly reduced in TPM2-knockout organoids, confirming efficient TPM2 suppression at the protein localization level. Vimentin staining was also reduced after TPM2 knockout, suggesting attenuation of an EMT-associated phenotype (Figure 6A). Quantitative analysis further confirmed significant decreases in TPM2-positive and Vimentin-positive areas in TPM2-knockout organoids compared with control organoids (Figure 6B−C; TPM2-positive area, control vs. TPM2KO: 52.86 ± 2.71% vs. 7.23 ± 0.72%, *P* < 0.001; Vimentin-positive area, control vs. TPM2KO: 15.75 ± 0.89% vs. 4.10 ± 0.43%, *P* < 0.001**)**.

Given that TPM2-high tumors were enriched in EMT- and Notch-related pathways in the preceding analyzes (Figure 4B−C), we next assessed whether TPM2 depletion affected Notch pathway activity and EMT/ECM-related transcriptional programs. RT-qPCR analysis showed that TPM2 knockout reduced the expression of Notch pathway-associated genes, including NOTCH1, HES1, and HEY1. In addition, EMT-associated markers, including VIM, SNAI1, and CDH2, as well as ECM-related genes, including FN1 and COL1A1, were downregulated after TPM2 depletion (Figure 6D). Consistently, western blot analysis showed decreased Notch1 and HES1 protein levels in TPM2-knockout organoids compared with non-targeting control organoids (Figure 6E).

Together, these findings suggest that TPM2 depletion is associated with reduced expression of Notch pathway-associated markers and EMT/ECM-related programs in ICC organoids. Based on these findings and the functional drug-response data, we propose a working model in which TPM2-associated cytoskeletal and ECM-related programs may contribute to Notch-EMT signaling and gemcitabine tolerance in ICC tumor cells (Figure 6F).

## Discussion

In this study, we identified TPM2 as a fibroblast-associated candidate linked to cell-matrix remodeling and gemcitabine response in ICC. TPM2 expression was associated with extracellular matrix remodeling, poor clinical outcomes, and activation of pro-tumorigenic signaling pathways. Functional studies in patient-derived organoids further demonstrated that TPM2 contributes to gemcitabine tolerance and is associated with EMT/ECM-related programs and Notch signaling. Collectively, these findings suggest that TPM2 may represent a candidate linking fibroblast-associated stromal remodeling with tumor cell plasticity and chemotherapy response in ICC.

A major feature of cholangiocarcinoma is its highly desmoplastic tumor microenvironment, characterized by abundant fibroblast infiltration and ECM deposition.^6^ ^,^ ^29^ This stromal structure is not only a physical scaffold, but also actively supports tumor progression, invasion, and therapeutic resistance.^6^ ^,^ ^9^ ^,^ ^29-32^ In recent years, single-cell and multi-omics approaches have provided important tools for dissecting tumor heterogeneity, microenvironmental remodeling, and evolutionary programs in biliary tract cancers.^16^ ^,^ ^33^ ^,^ ^34^ Consistent with these advances, our single-cell analysis revealed marked fibroblast enrichment and increased activity of tumor-related pathways within fibroblast populations (Figure 1). Moreover, hdWGCNA identified fibroblast-associated modules enriched in extracellular matrix organization and ECM-receptor interaction (Figure 2). These findings are in line with previous studies showing that fibroblasts and ECM remodeling are major determinants of ICC progression and therapeutic adaptation.^16^ ^,^ ^35^ Together, these findings support the relevance of fibroblast-associated ECM remodeling to tumor biology and chemotherapy response in ICC.

By integrating fibroblast-associated module genes with predicted gemcitabine sensitivity-related transcriptional changes, we further prioritized candidate genes potentially linking stromal remodeling with drug response. Among the overlapping genes, TPM2 and SOD3 were preferentially expressed in fibroblasts (Figure 3). However, TPM2 showed stronger clinical and biological relevance than SOD3 (Figure 4). TPM2 was more prominently upregulated in tumor tissues and was consistently associated with poorer disease-free and overall survival. Moreover, TPM2-high tumors exhibited enrichment of EMT, TGF-*β*, Notch, Wnt, cell-cycle, and DNA repair programs, whereas metabolic pathways were relatively suppressed. These results suggest that high TPM2 expression is associated with a less differentiated tumor state characterized by enhanced stromal, proliferative, and adaptive programs.

TPM2 belongs to the tropomyosin family of actin-binding proteins and is involved in actin filament stability and cytoskeletal organization.^24^ Cytoskeletal remodeling is closely linked to cell adhesion, contractility, migration, EMT, and ECM remodeling, which are central processes in tumor invasion and therapeutic adaptation.^25^ ^,^ ^36^ ^,^ ^37^ Therefore, the observed association between TPM2 and ECM/EMT-related programs is biologically plausible. Pseudotime trajectory analysis further demonstrated that TPM2 expression varies dynamically along inferred cellular states (Figure 4D−F), suggesting that TPM2 may be associated with dynamic cellular states rather than simply reflecting differences in stromal abundance. Although pseudotime analysis cannot establish causal ordering, these findings provide additional evidence for an association between TPM2 expression and cellular remodeling states in ICC.

A key strength of this study is the use of patient-derived ICC organoids to functionally evaluate the role of TPM2 in gemcitabine response. Previous studies have shown that biliary tract cancer organoids can retain key features of parental tumors and can be used for drug response assessment.^22^ ^,^ ^23^ In our study, organoids from two patients with advanced ICC preserved histological and lineage characteristics of their corresponding tumors and exhibited distinct responses to gemcitabine that were broadly consistent with clinical imaging outcomes (Figure 5A−D). Importantly, TPM2 knockout in the relatively gemcitabine-resistant ICCO2 model significantly enhanced gemcitabine-induced cytotoxicity, demonstrating a functional role of TPM2 in chemotherapy tolerance (Figure 5E−H). To further explore the molecular mechanisms underlying TPM2-mediated gemcitabine response, we investigated EMT/ECM-related programs and signaling pathways following TPM2 depletion in ICC organoids. TPM2 knockout reduced the expression of EMT-associated markers and ECM-related genes and was accompanied by suppression of Notch pathway activity at both transcriptional and protein levels (Figure 6A−E). These findings indicate that TPM2 depletion attenuates a coordinated transcriptional program involving EMT, ECM remodeling, and Notch signaling.

Mechanistically, these observations are consistent with established mechanobiology frameworks in which extracellular matrix remodeling and cytoskeletal tension regulate intracellular signaling through mechanotransduction processes, thereby controlling epithelial plasticity and EMT programs.^38-41^ ECM stiffness and actin cytoskeletal dynamics have been shown to drive transcriptional reprogramming through integrin-FAK-RhoA and YAP/TAZ signaling pathways.^39^ ^,^ ^42^ ^,^ ^43^ In addition, Notch signaling is increasingly recognized as a mechanosensitive pathway, where ligand endocytosis, cytoskeletal organization, and mechanical force regulate receptor activation and downstream transcriptional output.^44-46^ Given the established role of TPM2 in actin filament organization and cytoskeletal regulation,^24^ our finding that TPM2 depletion simultaneously reduced ECM/EMT-related programs and Notch activity is consistent with a model in which TPM2-associated cytoskeletal remodeling converges on a broader cell-matrix-mechanotransduction network to regulate tumor plasticity and gemcitabine response. Although these observations do not establish a direct causal hierarchy among cytoskeletal remodeling, EMT, and Notch signaling, they provide a mechanistic framework linking TPM2 to chemotherapy tolerance.

Importantly, TPM2 was initially identified through fibroblast-associated transcriptional programs, whereas functional validation was performed in tumor organoids lacking stromal components. Our data nevertheless provide several lines of evidence suggesting that fibroblast-associated TPM2 programs and tumor-cell-intrinsic TPM2 may converge on related downstream processes. First, TPM2 was preferentially expressed in fibroblasts in the single-cell dataset, and TPM2-associated co-expression modules were enriched in extracellular matrix organization and ECM-receptor interaction (Figures 1 and 2). These findings are consistent with the established role of fibroblasts and reactive stroma in ICC progression, tumor-stroma crosstalk, and therapeutic adaptation.^31^ ^,^ ^32^ ^,^ ^35^ ^,^ ^47^ ^,^ ^48^ Second, in bulk ICC tumors, high TPM2 expression was associated with EMT, TGF-*β*, Notch, and Wnt signaling programs, suggesting that the TPM2-associated phenotype extends beyond stromal abundance to tumor plasticity and signaling activation. Third, and importantly, TPM2 depletion in stromal-free patient-derived organoids reduced EMT- and ECM-related gene expression and suppressed Notch pathway activity, while enhancing gemcitabine sensitivity (Figures 5 and 6). Together, these observations raise the possibility that tumor-cell-intrinsic TPM2 can engage a subset of the molecular programs associated with fibroblast-driven ECM remodeling, even in the absence of stromal cells.

This interpretation provides a potential explanation for why TPM2 remained functionally relevant in a stromal-free organoid context. Rather than implying that tumor-cell TPM2 functionally replaces fibroblasts, we propose that fibroblast-associated and tumor-intrinsic TPM2 programs may converge on selected downstream consequences of cell-matrix and cytoskeletal remodeling. Such convergence is biologically plausible given the close coupling between actin organization, cell-matrix adhesion, mechanical signaling, EMT, and developmental pathways.^24^ ^,^ ^25^ ^,^ ^39-41^ In this framework, tumor-cell-intrinsic TPM2 may engage selected downstream features of a broader fibroblast-associated ECM-remodeling program, thereby maintaining cytoskeletal and signaling states that favor epithelial plasticity and chemotherapy tolerance. This interpretation remains a mechanistic hypothesis and does not establish a direct fibroblast-to-tumor effect.

This study has several limitations. First, the computational analyzes were based on publicly available single-cell and bulk transcriptomic datasets. The gemcitabine sensitivity in the TCGA cohort was predicted using oncoPredict rather than directly measured.^49^ Second, organoid validation was performed using a limited number of patient-derived models, and the TPM2 knockout experiments were focused on one relatively resistant organoid line. More ICC organoid models or cell lines would help confirm the general relevance of these findings. Third, conventional organoids do not fully recapitulate the stromal and immune components or the native extracellular matrix of the tumor microenvironment, limiting direct assessment of fibroblast-tumor cell interactions and matrix-dependent phenotypes.^50^ ^,^ ^51^ Therefore, although our organoid experiments demonstrate a tumor-cell-intrinsic role of TPM2 in gemcitabine tolerance, they cannot determine whether fibroblast-derived TPM2 directly contributes to tumor-cell chemotherapy resistance. Finally, although TPM2 depletion was associated with suppression of EMT and Notch signaling, the causal relationships among TPM2, cytoskeletal/ECM remodeling, Notch signaling, and gemcitabine response require further validation.

Future studies should use organoid-fibroblast co-culture systems, matrix-engineered organoid models, and spatial transcriptomic approaches to better define the stromal and tumor-intrinsic roles of TPM2. These models may also help clarify how TPM2-related cell-matrix remodeling affects drug penetration, tumor plasticity, and chemotherapy response.

## Conclusions

In conclusion, this study identifies TPM2 as a fibroblast-associated candidate linked to cell-matrix remodeling and gemcitabine response in ICC. Through integrated single-cell transcriptomic analysis, co-expression network modeling, clinical association studies, and patient-derived organoid validation, we demonstrate that TPM2 is associated with extracellular matrix remodeling, EMT-related transcriptional programs, poor clinical outcomes, and gemcitabine tolerance. Functional experiments further show that TPM2 depletion suppresses EMT/ECM-related programs and Notch signaling while enhancing gemcitabine sensitivity in patient-derived ICC organoids. Collectively, these findings support a model in which a fibroblast-associated TPM2 program is linked to cell-matrix remodeling, while tumor-cell-intrinsic TPM2 contributes to EMT/ECM-related programs, Notch signaling, and gemcitabine tolerance in ICC. Whether fibroblast-derived TPM2 directly contributes to tumor-cell chemotherapy resistance remains to be determined.

## Supplementary Material

Supplementary Material Supplementary figures.docx

## Data Availability

The public scRNA-seq datasets analyzed in this study are available in the GEO database under accession numbers GSE125449, GSE138709, GSE142784, and GSE181878. Bulk transcriptomic data were obtained from TCGA. Additional data generated during this study are available from the corresponding author upon reasonable request, subject to ethical and privacy restrictions.

## Abbreviations

ICC: Intrahepatic cholangiocarcinoma
hdWGCNA: High-dimensional weighted gene co-expression network analysis
ECM: Extracellular matrix
EMT: Epithelial-mesenchymal transition
HAIC: Hepatic arterial infusion chemotherapy
GEMOX: Gemcitabine plus oxaliplatin
GEO: Gene Expression Omnibus
PCA: Principal component analysis
UMAP: Uniform manifold approximation and projection
GO: Gene Ontology
KEGG: Kyoto Encyclopedia of Genes and Genomes
GDSC: Genomics of Drug Sensitivity in Cancer
GSVA: Gene set variation analysis
GSEA: Gene set enrichment analysis
MSigDB: Molecular Signatures Database
DFS: Disease-free survival
OS: Overall survival
DMSO: Dimethyl sulfoxide
PI: Propidium iodide
