# Supplementary material for: Fibroblast-associated TPM2 links cell-matrix remodeling to EMT-Notch signaling and gemcitabine resistance in intrahepatic cholangiocarcinoma
Source: Cancer Biol Ther. 2026 Sep 21;27(1):2725344. doi: 10.1080/15384047.2026.2725344 (PMC13596889; doi:10.1080/15384047.2026.2725344)
Supplement: Supplementary file 1 [file KCBT_A_2725344_SM0599.docx]

**Figure S1. Quality control, feature selection, and batch correction of single-cell RNA-seq data**

(A) Quality control metrics across cells, including the number of detected genes (nFeature_RNA), total RNA counts (nCount_RNA), mitochondrial gene percentage (percent.mt), and ribosomal gene percentage (percent.ribo).

(B) Relationships among sequencing depth and quality-control metrics. Left: correlation between nCount_RNA and nFeature_RNA. Middle: relationship between nCount_RNA and percent.mt. Right: relationship between percent.mt and percent.ribo.

(C) Identification of highly variable genes based on variance-stabilized expression.

(D) Elbow plot showing the variance explained by principal components.

(E) PCA plot colored by sample origin before batch correction.

(F) Harmony-integrated embedding showing sample distribution after batch correction.

**Figure S2. Sample-resolved and tissue-origin distributions of major cell populations**

(A) Relative contribution of individual samples to each annotated cell type, illustrating inter-sample heterogeneity in cellular composition.

(B) Relative proportions of cells derived from tumor and adjacent normal tissues within each annotated cell type. P values for differences in the distribution of tumor- and normal-derived cells were calculated using Pearson’s chi-squared test.

**Figure S3. KEGG pathway enrichment of fibroblast-associated module genes**

(A) KEGG pathway enrichment analysis of fibroblast-associated module genes performed using clusterProfiler. Bar length indicates gene count, and color represents adjusted P value.

**Figure S4. Expression patterns of additional candidate genes across cell populations**

(A) Feature plots showing the spatial distribution of EFEMP1, VIM, TPPP3, CYBA, FKBP10, and TMSB10 across cell populations. Color intensity indicates relative expression level.

**Figure S5. Expression patterns and clinical relevance of TPM2 and SOD3**

(A) Boxplots showing TPM2 and SOD3 expression in tumor and adjacent normal tissues from the TCGA cohort. Red indicates tumor tissues, and gray indicates adjacent normal tissues.

(B) Kaplan–Meier survival curves for DFS and OS comparing SOD3-high and SOD3-low groups.

(C) Expression dynamics of SOD3 along pseudotime.
